# Microscopy cell nuclei segmentation with enhanced U-Net

**DOI:** 10.1186/s12859-019-3332-1

**Published:** 2020-01-08

**Authors:** Feixiao Long

**Affiliations:** Hudongfeng Technology (Beijing) Co., Ltd., Sanjianfang South No.4, DREAM 2049 B05, Chaoyang District, Beijing, China

**Keywords:** Cell and cell nuclei segmentation, Deep learning, Enhanced U-Net

## Abstract

**Background:**

Cell nuclei segmentation is a fundamental task in microscopy image analysis, based on which multiple biological related analysis can be performed. Although deep learning (DL) based techniques have achieved state-of-the-art performances in image segmentation tasks, these methods are usually complex and require support of powerful computing resources. In addition, it is impractical to allocate advanced computing resources to each dark- or bright-field microscopy, which is widely employed in vast clinical institutions, considering the cost of medical exams. Thus, it is essential to develop accurate DL based segmentation algorithms working with resources-constraint computing.

**Results:**

An enhanced, light-weighted U-Net (called U-Net+) with modified encoded branch is proposed to potentially work with low-resources computing. Through strictly controlled experiments, the average IOU and precision of U-Net+ predictions are confirmed to outperform other prevalent competing methods with 1.0*%* to 3.0*%* gain on the first stage test set of 2018 Kaggle Data Science Bowl cell nuclei segmentation contest with shorter inference time.

**Conclusions:**

Our results preliminarily demonstrate the potential of proposed U-Net+ in correctly spotting microscopy cell nuclei with resources-constraint computing.

## Background

Cell or cell nuclei segmentation is typically the first critical step for biomedical microscopy image analysis [1]. On the basis of accurate cell or cell nuclei segmentation, multiple biological or medical analysis can be performed subsequently, including cell type classification [2], particular cell counting [3], cell phenotype analysis [4] etc., providing valuable diagnostic information for doctors and researchers. Although conventional image processing techniques are still employed for this time and labor consuming task, they often cannot achieve the optimized performance due to multiple reasons, such as limited capability of dealing with diverse images [1].

With the rapid developments of deep learning (DL) based techniques, multiple researchers begin to investigate the potential applications to employ DL in cell or cell nuclei segmentation. For example, C. Hernández et.al. [5] proposed using feature pyramid network (FPN) combined with VGG-style neural nets to predict the masks of cells. F. Araújo et.al. [6] employed convolutional neural network (CNN) to predict the highly abnormal cell regions in Pap smear test. T. Tran et.al. [3] used Seg-Net to segment white blood cells (WBCs) and red blood cells (RBCs) in peripheral blood smear images, showing 89.45*%* global accuracy. Among multiple cell or cell nuclei segmentation algorithms, U-Net [7] based ones are the most popular selections and often achieve the state-of-the-art segmentation results as reported in [1]. For example, R. Hollandi et.al. [8] proposed a method which employed both Mask R-CNN and U-Net to predict the segmentation of cell nuclei and the accuracy of algorithm outperformed 739 methods submitted to 2018 Kaggle Data Science Bowl. The first place algorithm on the same contest [9] also employed U-Net as fundamental model.

Although DL algorithms has demonstrated the efficiency in segmenting cell or cell nuclei, these state-of-the-art segmentation algorithms rely heavily on complex operations or strategies, such as model ensemble, bagging techniques or complicated pre- and post-processing of images, which impede the applications of algorithms in real clinical situations (often only equipped with limited computing power). For example, methods reported in [10] employed 2 U-Nets to make final predictions, increasing the accuracy at the sacrifice of higher computing load during inference. In addition, the wide spread use of simple bright- or dark-field microscopy can generate large number of images which need to be processed and analyzed rapidly. This requires the segmentation algorithms to be highly accurate and light-weighted, with the ability of running fast with constrained computing resources, since transferring large number of images to cloud server will generally cause long uploading time and large network pressure [11]. Hence, it is still necessary to develop new DL based technology to increase the accuracy of segmentation, especially with limited computing power.

Therefore, our aim in the manuscript is to explore a) the possible performance improvement of cell nuclei segmentation algorithms through DL with less pre- and post-processing of images; b) the possibility to deploy the model with limited computing power (in other words, the model size should be small). Considering our aim, several prevalent, winning methods in various biomedical competitions are to be excluded, such as multiple models ensemble or combination as mentioned above. Also for the same reason, deeper and complex models such as ResNet-101 [12], InceptionResnet-V2 [13] etc. working as backbone models are not preferred solutions. The possible solution is then to check if modifications of classical U-Net will boost the accuracy of segmentation as [14–16] shown. Therefore, in our methods, the encoded branch of conventional U-Net was redesigned to fuse more image features between shallow and deep hierarchy layers (see “Model structure” section). We argue that more fine-grained feature fusions from shallow layer will carry more details of semantic information, which will benefit the cell segmentation. In order to test the effect of the proposed structure (named as U-Net+), a series of experiments was performed (see “Results” section) with U-Net and U-Net++ [14] served as control. In the experiments, U-Net was employed as a benchmark of segmentation due to its wide application in such tasks. The selection of U-Net++ as control is based on the following considerations: in [14], it is reported that the model prediction results were based on the same cell nuclei segmentation dataset (see “Cell nuclei dataset” section) in our experiments. Besides, the comparisons between U-Net++ and other popular U-Net based structures were drawn and U-Net++ was reported to achieve the best results.

However, performing impartial comparisons between different DL algorithms may not be as straightforward as anticipated. First, it is widely acknowledged that without knowing the details of training, such as optimization methods, training related parameters (e.g. initial learning rate, learning rate decay scheme etc.), the prediction results of deep model can be quite difficult to reproduce. To make things worse, implementations of deep models with different DL frameworks can also induce the variations in accuracy, running time [17, 18] etc. In other words, one prerequisite condition for impartial model comparisons is to employ the same DL framework with comparative training settings and scheme. Otherwise one cannot discriminate the origin of metric value variations. Second, for deep models, sometimes it will be difficult to accurately identify the source of empirical gains either from the modification of model or just the results of fine tuning the parameters [19]. For example, research from [20] has shown that with adequate settings of hyper-parameters (batch size, image patch size and number of pooling operations), classical U-Net without complex structure modifications can achieve state-of-the-art performance on several challenges or dataset. Third, different image pre- and post-processing techniques can lead to the changes of predictions. Based on above reasons, all the other factors (pre- and post-processing, training scheme, hyper-parameters settings, number of filters in each depth etc.) except the neural nets structure were fixed in our experiments, ensuring that the performance changes were indeed originated from different design of model structure.

The main contributions of our manuscript are: with strictly controlled single variable (neural net structure) experiments, the proposed U-Net+, which can be viewed as a light-weighted enhanced U-Net (through implementing densely connected convolutional blocks between different depth in encoded branch), has been proved to have comparative performance with other popular U-Net like structures at least in cell nuclei segmentation task.

The manuscript is arranged as following. In “Results” section, the comparisons of model performances with different settings are introduced with details. Some necessary explanations of results are also demonstrated in the same section. Some critical problems when employing DL techniques in analyzing biomedical images are discussed in depth in “Discussions” section. Moreover, future improvements of our work will be illustrated in “Conclusion” section. In “Methods” section, the details of cell nuclei dataset are introduced. The techniques employed in pre-processing of images and how to generate the training and validation set will also be described. The structure of U-Net+ and intuitive explanations of the design will be emphasized and illustrated in the same section. Besides, the details of training settings will be introduced, confirming that all the comparisons of algorithms are founded on exactly the same base. At the last part of “Methods” section, the post-processing and metric values to evaluate the performance of algorithms are illustrated.

## Results

In the experiments, the number of filters in the first layer *B* was set to 8 and 16 respectively and the model depth *N* was fixed to 5 (see “Model structure” section for more details). Note that these settings would make U-Net and U-Net++ with fewer number of weights compared to their original published version. Besides, to U-Net++ model, when *B*=16, the regularization parameter *λ* was set to 0.0001 as suggested in [14] and 0.0 when *B*=8.

### Validation loss curve during training

**Fig. 1:**
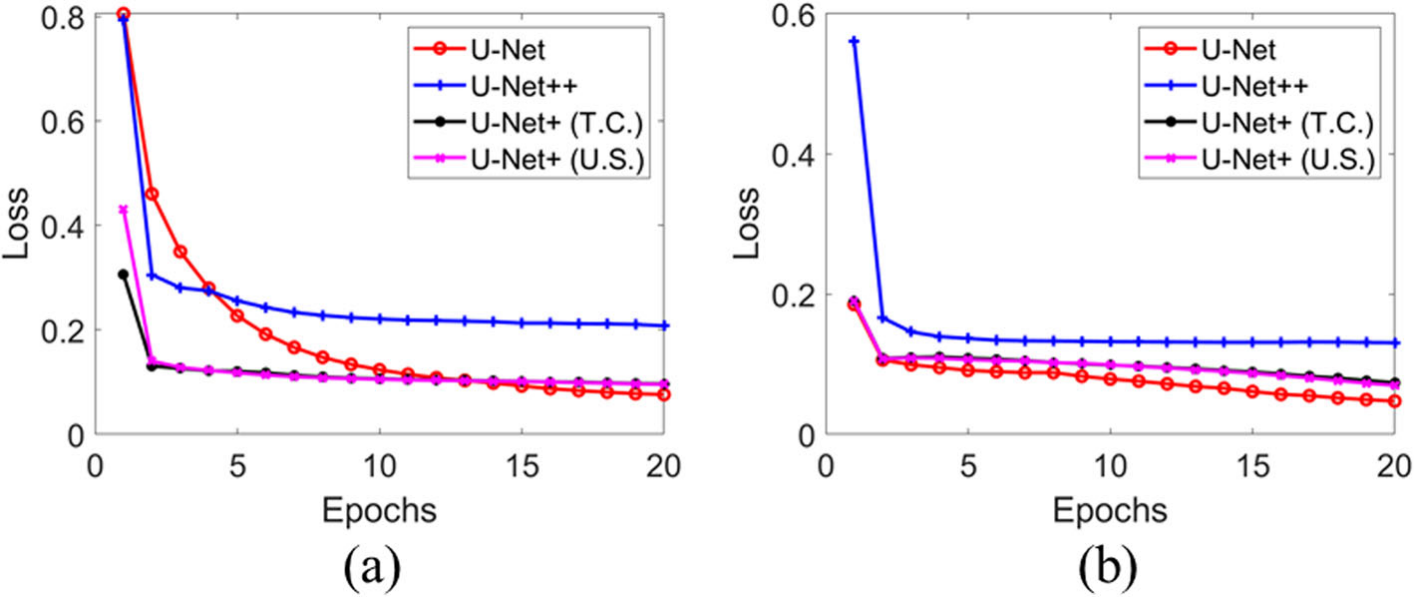
Validation losses of U-Net, U-Net++ and U-Net+. **a** *B*=8. **b** *B*=16

First, the validation losses of the models with different *B* settings during training are shown in Fig. 1. To U-Net+, T.C. denotes transposed convolutional and U.S. represents up-sampling operation (see “Model structure” section for explanations). From the figure, it is clear that the validation losses of all the models remain stable after first 20 epochs training. At the end of training, the validation losses of U-Net and U-Net+ approach similar values around 0.13 to 0.15 for both *B*. Also, different up-sampling manners of U-Net+ make no obvious changes of validation loss at the end of training. In contrast, in both cases, the validation losses of U-Net++ are still higher than losses of the other two models, which encourages us to perform fine tuning of U-Net++ for another 5 epochs as stated in “Training settings” section. By the end of the fine tuning, the validation loss of U-Net++ reaches similar values (0.17) as U-Net and U-Net+, meaning all models have been trained with similar extent. This also demonstrates that comparisons of model performance are firmly based on the same ground.

### Segmentation with *B*=16

**Fig. 2:**
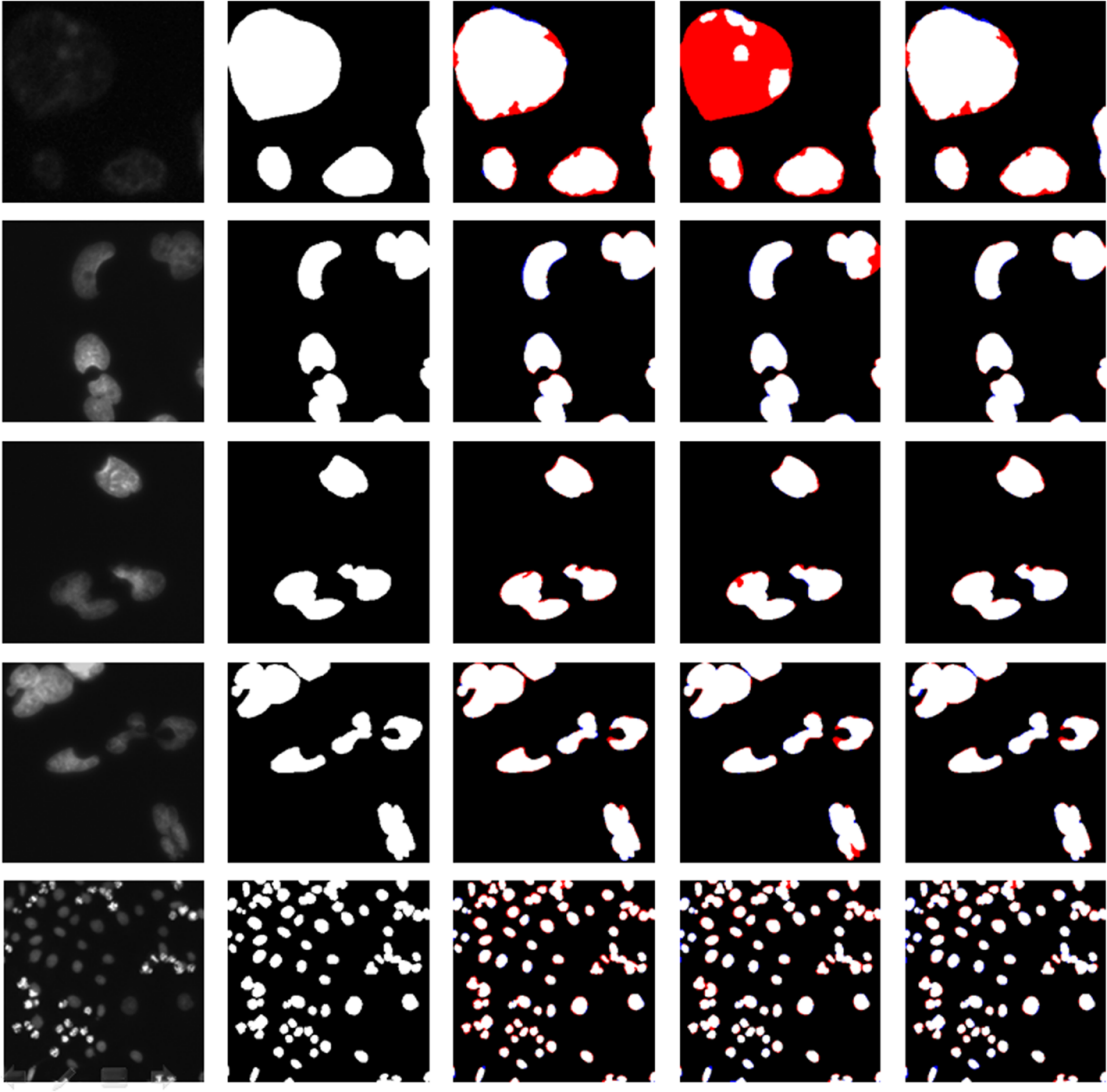
Segmentation comparisons between U-Net, U-Net++ and U-Net+. Red color indicates the FN predictions while blue color shows the FP predictions. From left to right, the columns represent original image, ground truth, U-Net predictions, U-Net++ predictions and U-Net+ predictions respectively

The comparisons of segmentation results (image size 256×256) with different model structures (U-Net+ with transposed convolutional operation) are shown in Fig. 2. The images in first 4 rows were directly cropped from the original larger ones. In the figure, the white color shows the correct predictions. In order to clearly see the difference between the predicted cell label and ground truth, two colors are employed: the red color shows the FN predicted pixels (pixels in ground truth but not in prediction) and blue one indicates the FP predicted pixels (pixels in predicted label but not in ground truth). From the figure, to most cases, the error just occurs at the boundaries of cell nuclei. Clearly, the U-Net+ predictions exhibit the least amount of colored pixels, showing the highest accuracy.

Table 1 shows the comparisons of average IOU, precision and average inference time (ms) per patch (256×256, see “Training and validation set generation” section) in the test set. In Table 1, the average IOU is reported as mean value ± standard deviation. The meanings of T.C. and U.S. are the same as shown in “Validation loss curve during training” section. Besides, the number of weights for each model is reported in the table with unit million (M). From the table, the average IOUs of U-Net+ (T.C. and U.S.) and U-Net++ are similar with difference less than 1.5*%* although metric values of U-Net+ are better, but these values exceed the average IOU of U-Net more than 2%. The average precision also shows the same trend as average IOU. We observe that U-Net+ with transposed convolutional operation achieves better performance than U-Net+ with up-sampling operation, probably because of its more number of weights (better capability of generalization). However, when comparing the number of weights of different models, U-Net+ has greater advantages than U-Net++. From Table 1, the number of weights of U-Net+ with transposed convolution is about 15% less compared with U-Net++ model, which only needs less than 8 Mb space to save. The number of weights of U-Net+ with up-sampling is even fewer, only 75% of the number of parameters in U-Net++ model. The number of weights of U-Net+ (T.C. and U.S.) is slightly fewer than U-Net. The average inference times of U-Net+ with transposed convolutional operation and U-Net are almost the same (approximately 15 ms), which are obviously less than the average inference time of U-Net++ (22 ms). However, the average inference time of U-Net+ with up-sampling is almost the same as U-Net++ although with fewer number of weights. The difference of inference time between two up-sampling modes of U-Net+ is because when performing the transposed convolution, the number of channels in output tensor has already been reduced to half of input tensor before performing subsequent two convolutional operations. Therefore, this will decrease the size of the kernel of subsequent first convolutional operation and reduce the number of floating-point computations. In contrast, the number of channels in output tensor with up-sampling manner remains the same as input, hence increasing the total computation time. In sum, to *B*=16, U-Net+ performs better than the other two competing models due to more accurate predictions, less number of weights and lower inference time.

**Table 1:** Comparisons of model performances with *B*=16

	U-Net	U-Net++ ^∗^	U-Net+ (T.C.)	U-Net+ (U.S.)
Ave. IOU (*m*±*s*)	0.533±0.173	0.552±0.217	0.566±0.178	0.551±0.187
Ave. precision (%)	60.0	61.7	63.4	62.0
No. of weights (M)	1.941	2.262	1.926	1.730
GPU infer. time (ms/patch)	14.9	22.0	15.3	21.5

* regularization parameter *λ*=0.0001

### Segmentation with *B*=8

Table 2 lists the similar metrics for evaluating the accuracy of segmentation with *B*=8. The performances of U-Net+ and U-Net++ are similar (although U-Net+ is slightly better in average IOU than U-Net++) and exceed the performance of U-Net a lot (over 3% for average IOU and average precision). The overall performance of *B*=8 almost remains the same compared to the case *B*=16 for U-Net++ and U-Net+. In contrast, the performance of U-Net decreases a little. From Tables 1 and 2, we emphasized the pivotal role of regularization parameter in training U-Net++, which influences the accuracy a lot on test set.

**Table 2:** Comparisons of model performances with *B*=8

	U-Net	U-Net++ ^∗^	U-Net+ (T.C.)	U-Net+ (U.S.)
Ave. IOU (*m*±*s*)	0.526±0.170	0.564±0.181	0.572±0.179	0.567±0.177
Ave. precision (%)	59.5	63.0	62.5	62.4
No. of weights (M)	0.486	0.566	0.484	0.435
GPU infer. time (ms/patch)	9.5	14.1	12.4	14.0

* regularization parameter *λ*=0.0

One possible speculation of this phenomenon is the number of weights which determines the capacity of deep models. In order to verify this hypothesis, several more experiments were performed and the results are shown below. When *B*=16, U-Net++ model was also trained using regularization parameter *λ*=0.0 with other training settings same as *λ*=0.0001. We observed the performance decreasing with this setting. The average IOU is only 0.503±0.217 and average precision is 57.1*%*. Therefore, in this case *B*=16, we argue that larger number of weights of U-Net++ is prone to over-fit on training set and non-zero regularization parameter reduces the over-fitting, thus increasing the accuracy of the U-Net++ on test set. In contrast, when *B*=8, setting non-zero regularization parameter will restrain the capacity of U-Net++ and intuitively speaking, this will make deep model not be able to cope with complex training and test sets, also leading the reduced performance (average IOU 0.503±0.162 and average precision 57.1*%*).

From Table 2, the average inference times of U-Net+ with transposed convolution and U-Net are less than average inference time of U-Net++ while the average time of U-Net+ with up-sampling is still similar as U-Net++. However, the average inference time of U-Net (9.5 ms) is much less than the average one of U-Net+ with transposed convolution (12.4 ms). Herein, we speculate that more concatenation operations of U-Net+ and U-Net++ will lower the inference speed compared to U-Net. However, the actual reason needs to be explored in our future work.

## Discussions

In the manuscript, we proposed an enhanced version of classical U-Net, through re-designing the encoded branch with densely connected convolutional blocks. Through strictly controlled experiments, the performance improvements of U-Net+ are confirmed from the new design of model structure. Overall, U-Net+ performs more accurate segmentation with fewer number of weights compared with widely employed U-Net and state-of-the-art U-Net++ in the first stage test set of cell nuclei segmentation contest (grey-scale images only). Here we would like to point out that U-Net++ with deep supervision training during inference (accurate mode) would probably achieve better performances than U-Net+ reported in the manuscript. Briefly, as mentioned above, since U-Net++ has multiple up-sampling branches (*L*^1^ to *L*^4^ [14], in contrast, multiple down-sampling pathways exist in U-Net+), when performing accurate mode prediction, it actually averages outputs from different up-sampling branches and eventually increases the accuracy of prediction. However, under that circumstance, U-Net+ still has advantage of 15 to 25% fewer number of weights and shorter inference time as shown in “Results” section. Note that another advantage of the model with fewer number of weights allows setting larger batch size during training under certain hardware configurations, which will reduce the total time of training procedure with specific number of training epochs. On the other hand, when performing the fast mode prediction through model pruning, U-Net++ utilizes one output form multiple up-sampling branches. In fact this can be viewed as one U-Net with smaller *N*, herein decreases the inference time paying the price of moderate accuracy loss. Since the prediction accuracy of U-Net+ is better than accuracy of segmentation inferred by branch *L*^4^ of U-Net++, it can be concluded the results from U-Net+ will still be superior to predictions from up-sampling branches *L*^1^ to *L*^3^. Even if the inference time of segmentation branch other than *L*^4^ may be shorter than U-Net+ and U-Net, we emphasize that the comparison may not be neutral because the shorter inference time is due to smaller number of layers (fewer floating-point operations) than U-Net or U-Net+.

From the aspect of DL techniques, there still exist quite a lot open theoretical problems. For instance, without theoretical support, it is impossible to understand why some U-Net based structures will improve the accuracy of segmentation, as questioned by [19]. In the manuscript, we can only provide intuitive explanations without any theoretical explanations while this phenomenon also appears commonly in most biomedical image analysis papers using DL. Large amount of novel neural nets design emerge, yet the efficacy is only verified with the experiments not theory. Besides, because of the deficiency theoretical support of tuning hyper-parameters, in most cases, it will be quite difficult to validate whether the trained model is optimized. For example, if continuously fine tuning the parameters of all the models examined in the manuscript, is it still possible to acquire better segmentation? The answer would be quite probably positive. However, the correct direction for adjustments may not be easily discovered without theoretical guidance. Hence, it urgently needs rigorous theoretical support for DL further development. From the viewpoint of applications, although U-Net+ has the potential to deal with complex cell image segmentation, there still exist lots of unsolved problems when utilizing such DL techniques in real clinical situations, which actually impede the extensive applications of DL techniques in biomedical image analysis.

First, the clean, well-organized data in almost all the biomedical related contests is an ’optimized’ or probably ’over-optimized’ version of real data which could contain noise, artificial damages, huge staining non-uniformity, large variability of peripheral illumination or various artifacts etc. In other hand, accurate annotation of medical related data requires highly professional prior knowledge and is often time consuming. To make things worse, it is quite common to observe human annotation error in the dataset even for professional annotators, which will dramatically influence the accuracy of models. All factors mentioned above will make these open medical datasets not only contain annotation error [21] but also insufficiently reflect multiple characters of real data. The DL models pre-trained on this kind of dataset can be anticipated to have deteriorated performance when employed in clinical institutions. The limited performances of DL techniques under real situations explain why conventional image processing techniques are still prevalent in area of biomedical image analysis [22], which often do not require large number of correctly annotated samples to train the model. Second, the researchers from the DL community are continuously pushing forward the performance of deep model, even with very complicated combinations of various techniques, hoping to get state-of-the-art metric values, such as IOU or classification accuracy. For instance, [8] shows a complicated training and inference method to increase the average precision of cell nuclei segmentation. In other tasks of biomedical image analysis, the phenomenon with more and more complicated methods is also commonly seen. However, one crucial question has to be asked, does the increase of metric values of deep model really mean the significant improvements of clinical issues? The answer may differ from the application to application [21]. Hence, whether DL techniques indeed increase the accuracy and efficiency of solving real biomedical relevant issues has to be carefully examined, which is also the next step of our future work to test the efficacy of proposed U-Net+ in clinical cases. We also would like to point out another disadvantage of employing complicated DL models, which often leads higher model loading burden and longer prediction latency, is its difficult or even infeasible deployment in clinical institutions.

## Conclusion

In the manuscript, the model performance of proposed U-Net+ achieves approximately 1.0*%* to 3.0*%* gain with fewer number of weights and shorter inference time compared to U-Net and U-Net++ in the first stage test set of 2018 Kaggle Data Science Bowl cell nuclei segmentation contest (grey-scale images only). Our experiments preliminarily demonstrate a potential application of U-Net+ in microscopy cell image segmentation. However, besides theoretical explorations, there still exists much work to be done to bridge the gap between DL based segmentation and clinical applications as discussed above. In the future, from the aspect of proposed model structure, the generality of U-Net+ must be fully validated by multiple segmentation tasks, such as lung or liver segmentation from different imaging modalities (X-ray or MRI). Besides, cell nuclei dataset consisting of much more samples with various capture conditions has to be established to further train U-Net+ and test the usability. Moreover, the performance of U-Net+ with edge computing will also be appraised in our future work.

## Methods

### Cell nuclei dataset

**Fig. 3:**
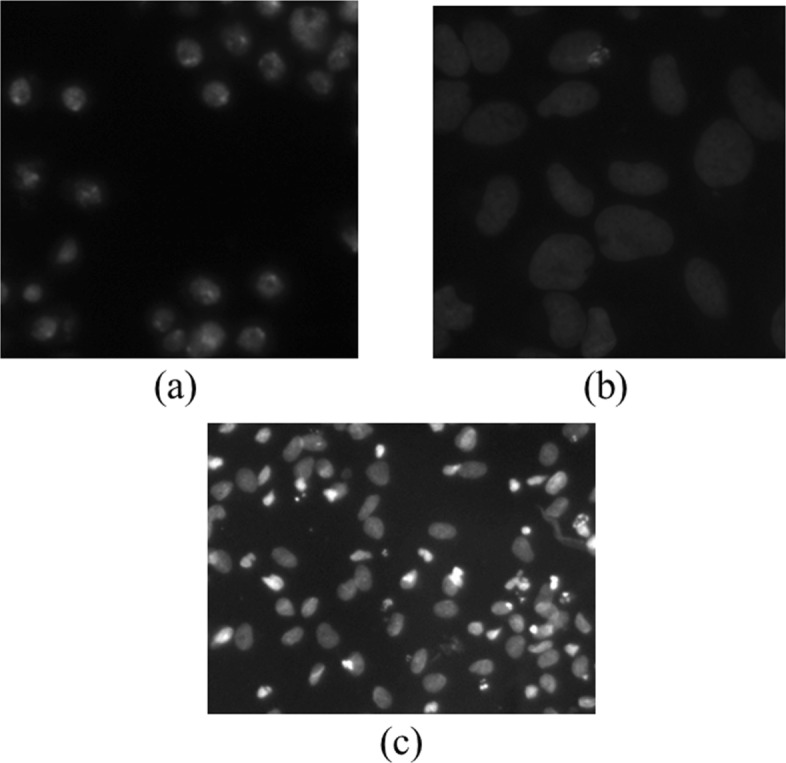
Original cell images. Different cell appearance (**a**), background illumination (**b**) and microscopy magnification (**c**) are shown

The dataset from 2018 Kaggle cell nuclei segmentation competition [23] was employed as original training and test dataset. The dataset itself is extremely challenging for the images were captured with various situations, such as different cell type, illumination status, imaging modality (bright-, dark-field and fluorescence microscopy) and image size etc. As shown in the Fig. 3a and c, it is obviously seen that the cell appearance is different. Besides, the illumination status is nonuniform between different samples, making nuclei detection and segmentation more challenging. Without contrast adjustment, one could hardly observe the details and boundaries of cells shown in Fig. 3b. The released first stage training set consists of 670 cell images accompanied with their segmentation stored as image files. In contrast, the first stage test set consists of 65 images with the segmentation encoded as run-length files (check the contest for more information). Considering bright- or dark-filed microscopy is widely employed in clinical institutions and most samples in the released dataset belong to these kinds of microscopy images, only the grey-scale images were employed to be investigated in our experiments. This leads to the training set with 546 images and the test set with 53 images accordingly. To eliminate or reduce the non-uniform illumination noise, several image pre-processing techniques had to be employed and will be illustrated in the next subsection.

### Images pre-processing

**Fig. 4:**
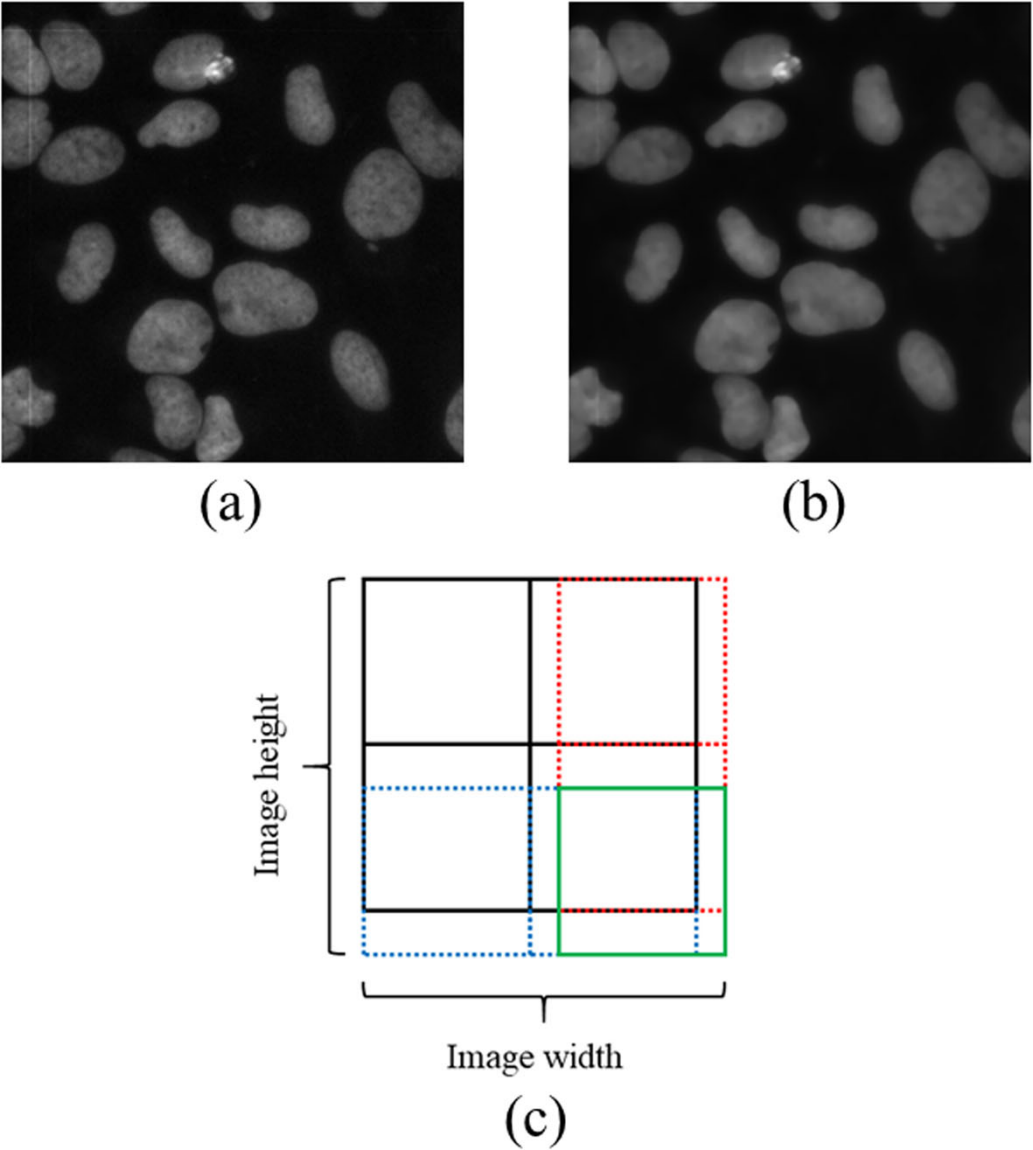
Demonstration of pre-processing images and patches generation pattern. **a** CLAHE processed image. **b** Total-variation de-noised image. **c** Patches generation pattern. Each small rectangle represents one patch

In order to adjust the contrast of images in the dataset, especially for dimmed ones as shown in Fig. 3b, contrast limited adaptive histogram equalization (CLAHE) [24] algorithm was first employed. However, for some images in the training set, CLAHE will also increase the magnitude of image noise and possibly make the model ’focus on’ these amplified high frequency noisy parts. Herein, total-variation de-noising [25] algorithm was subsequently applied to the histogram equalized image to reduce the noise and smooth the image yet with cell edge preserved. Finally, the value of the image was rescaled to [0,1] conventionally. Figure 4a and b shows the processed copy of Fig. 3b. It is visually clear that the image details, such as boundaries, can be easily seen compared with original dimmed one.

### Training and validation set generation

Normally, it is necessary to augment the number of training samples to reduce over-fitting of the deep model and mimic possible environmental changes during the capture of images, such as deficient background light intensity, image blurring due to motion and staining/color non-uniformity etc., which may not be sufficiently represented in original dataset. Considering the dataset with great variation of illumination intensity, the augmentation techniques (besides common geometry augmentations) adjusting the hue and contrast or illumination of images were mainly employed. Also, out of focus of microscopy and the de-noising pre-processing operation would result in relative blurred edges of cells, which led us to pay attention to various blurring augmentations. To sum, several image augmentations, including blurring (Gaussian, median and average), hue, color saturation and contrast adjustments, were randomly applied to the original images from dataset. Here we would like to point out that our image augmentations are indeed quite light compared to some public winning schemes of the contest such as [9] shown.

After the images were augmented and pre-processed, multiple patches with target size 256×256 were extracted from single image followed by the rules described below. In details, a structured grid (*iΔx*,*jΔy*) with grid spacing *Δx*=*Δy*=256 along column and raw was first generated and the patches whose coordinates of top-left corner coincide with structured grid point were cropped from the image accordingly (see Fig. 4c black blocks). For the images with height or width less than 256, zero-padding was applied to the corresponding dimension to make the cropped patches always with the same target size as others. Besides, to the images whose height or width is larger than 256 but indivisible by 256, patches were re-cropped at the boundary of image to ensure whole image was covered (Fig. 4c red, blue dotted and green solid squares). In addition, random selected patches with same target size were cropped from the image to further increase the number of training samples. To each randomly cropped patch, the ratio between foreground (nuclei) and background must be greater than a pre-defined threshold (0.4 in the experiments), otherwise the patch would be discarded. The generated patches were then randomly split into two subsets, training and validation set with proportion 8:2.

### Model structure

**Fig. 5:**
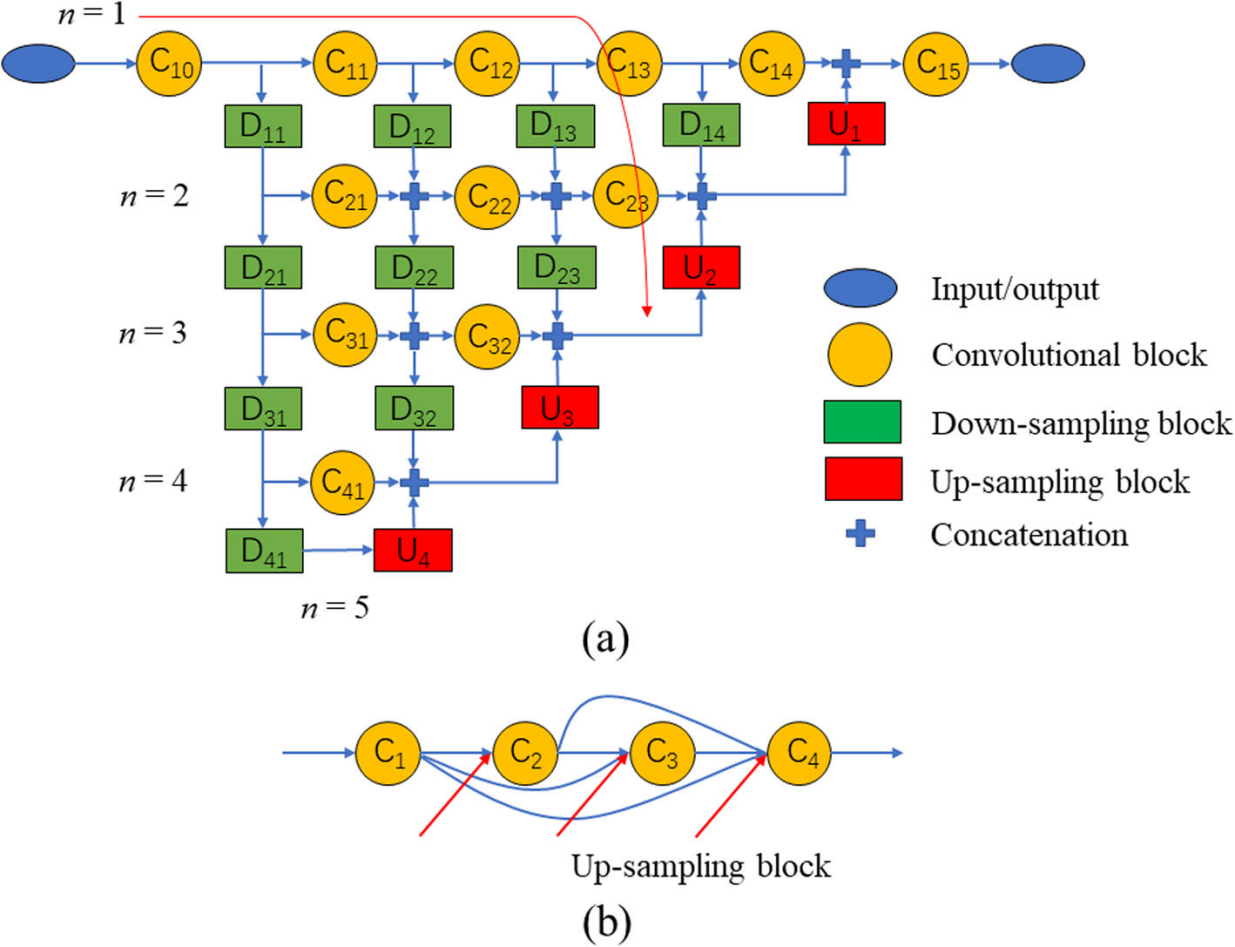
Structure of U-Net+ and one skip-connection from U-Net++. **a** Structure of U-Net+. For simplicity, the convolutional blocks are not shown in the up-sampling blocks. **b** One skip-connection of U-Net++. Re-drawn from [14] Fig. 1b

Inspired by the classical structures of U-Net [7] and U-Net++ [14], we redesigned the encoding branch of U-Net as shown in Fig. 5a. Here are several key observations and explanations of the proposed U-Net+ structure. First, at the same U-Net+ level (depth), compared to direct concatenation between encoded and decoded features as U-Net, the image (encoded) features of U-Net+ are generated by several more convolutional operations to get more abstract semantic representations (e.g. Fig. 5a, *C*_12_,*C*_13_). Second, features from shallow level have more pathways to be merged and convoluted with subsequent encoded features of deeper levels before decoded by transposed convolutional or up-sampling operations. Intuitively, before up-sampling, compared with U-Net or U-Net++, the encoded features will be enriched with more features from low level, fine-grained and high-resolution images, increasing the accuracy predicting the boundary of objects. Besides, the fusion of low resolution image features from deeper layers, which are recognized to carry more context information [26], also makes U-Net+ increase the accuracy of segmentation. For instance, the input of *U*_2_ up-sampling block will not only consist of the output of deeper up-sampling block *U*_3_ and encoded image features from the same level *n*=3, but also directly concatenate the encoded image features from level 1 and 2 (see the arrow in Fig. 5a, showing one possible image features flow, *C*_10_→*C*_11_→*C*_12_→*D*_13_→*D*_23_→*U*_2_) which are not encoded and decoded by the deeper parts of neural net. Also note that the image features flow as well as the gradient flow during back-propagation are different compared with U-Net++ model, which can be viewed as an ensemble of multiple U-Nets with distinct depth due to multiple up-sampling branches [14] (see Fig. 5b).

Specifically, in each convolutional block (*C*_11_,*C*_23_ etc.), convolutional operation was performed with kernel size 3×3, stride (1,1) and zero-padding as most modern structures used. The batch normalization layer [27] was employed for accelerating the training speed and the activation function for each neuron in the block was set to rectified linear unit (ReLU). The down-sampling block was implemented through the convolutional operation with stride (2,2) in row and column dimension respectively. The rest settings of down-sampling block were the same as convolutional block (kernel size, batch normalization and activation function). The up-sampling block was implemented by either transposed convolution (kernel size 3×3 and stride (1,1)) or up-sampling (repeating tensor rows and columns, such as TensorFlow UpSampling2D) followed by two convolutional blocks (not shown in Fig. 5a for simplicity). The difference of performance between two up-sampling manners is illustrated in “Results” section. Note to a particular operational block, for instance *C*_23_ or *U*_1_, the inputs were the concatenation of all the connected block outputs (see Fig. 5a). With depth increasing, the number of filters in each convolutional, down-sampling or up-sampling block was accordingly set to 2^*n*−1^*B*,*n*=1,...,*N*, in which *B* represents the number of filters in the first level and *N* denotes the number of levels or depth (in Fig. 5a, *N*=5). For example, the number of filters in convolutional block *C*_31_,*C*_32_ were all 4*B* while the number of filters in the down-sampling block *D*_41_ was set to 16*B* (this also explains why *N*=5 in the figure). Both *B* and *N* determine the size of U-Net+. For increasing the computational efficiency, *B* was set to the multiplier of 2. To the convolutional block *C*_15_, only single convolutional operation (1×1 kernel and stride (1,1)) with sigmoid activation function was employed so as to predict the probability of each output class. In nuclei spotting and segmentation, the number of classes was 1, indicating the cell nuclei.

Another point needs to be noticed is the fewer number of weights of U-Net+ compared with U-Net++ (also see “Results” section for detailed comparisons). This can be concluded from different skip-connection structure between U-Net+ and U-Net++ since down-sampling and up-sampling branches of two structures are similar. In Fig. 5b, to U-Net++, the input of each convolutional unit is the concatenation from two categorical outputs; the tensors from previous densely connected blocks along the skip pathway at same depth and the ones from up-sampling blocks located in the deeper level. This will enlarge the size of input tensor before performing convolutional operation especially for the convolutional blocks near the decoded branch (e.g. *C*_3_ or *C*_4_ shown in Fig. 5b) and herein increase the number of channels of convolutional kernels and model weights. For simplicity, assuming the number of channels of output tensor for every convolutional block in Fig. 5b is *c* and the number of channels for up-sampling block is *b* (in [14], *b*=*c*), then to convolutional block *C*_4_, the kernel size is (3*c*+*b*)×*h*×*w*×*c*, in which *h* and *w* represent the kernel height and width respectively. In contrast, to U-Net+, the inputs to most convolutional blocks (e.g., *C*_22_,*C*_32_ etc.) only consist of two outputs from connected convolutional and down-sampling blocks. For instance, using the same assumptions and notations as U-Net++, the kernel size of convolutional block *C*_23_ is only 2*c*×*h*×*w*×*c*. Besides, the kernel size remains the same for all the intermediate convolutional blocks at same depth. Overall, this design results in less number of weights of U-Net+ than U-Net++.

### Training settings

As illustrated in “Background” section, the parameters besides the neural net structure were all fixed during training for subsequent rigorous comparisons of performance of different model. Below lists the details during training models (U-Net 1, U-Net+ and U-Net++ 2).

The loss function is set to negative log Dice coefficient loss plus binary cross-entropy loss between the predicted and true labels of all training samples, namely,

1 $$ \mathcal{L} = \lambda_{\text{BCE}} \mathcal{L}_{\text{BCE}} + \frac{1}{M}\sum_{i = 0}^{M}-\log D\left(y_{\text{true}}^{i}, y_{\text{pred}}^{i}\right)  $$

In Eq. 1, *λ*_BCE_ is employed to balance log loss and binary cross-entropy loss and empirically set to 1.0 in the experiment. The Dice coefficient is defined as,

2 $$ D(y_{\text{true}}, y_{\text{pred}}) = \frac{2|y_{\text{true}} \cap y_{\text{pred}}|}{|y_{\text{true}}| + |y_{\text{pred}}|}  $$

Here, |·| denotes cardinal operator. Geometrically, Dice coefficient measures the similarity between 2 sets (including size and position). During training, Adam algorithm [28] with initial learning rate 0.0005 was employed to optimize the loss function defined in Eq. 1. In order to reduce the over-fitting of deep model, loss was monitored on the validation set. The model with best accuracy (lowest loss) would be stored, employed to predict the segmentation on the test set. The batch size was adjusted based on the type and size of the model but to saturate all the GPU memory in the experiments inspired by [20]. The number of epochs was empirically set to 20 and the losses on the validation set were stable (see “Validation loss curve during training” section) at the end of training. Here several points need to be noticed when training U-Net++. First, in the experiments, U-Net++ was fine-tuned for another 5 epochs on the basis of first 20 epochs training results with smaller learning rate 0.0001 or 0.0003 due to its lower convergent rate. Second, we found *L*_2_ regularization played important role of U-Net++ performance, possible due to the larger number of weights of U-Net++ (see“Segmentation with *B*=8” section for the discussions in details). Third, the deep supervision training of U-Net++ which may increase the accuracy of predictions during inference with accurate mode was not employed since no corresponding structures exist for both U-Net and U-Net+. Indeed, we emphasize that, in the manuscript, the comparison between results predicted by U-Net++ *L*^4^ segmentation branch and corresponding predictions from U-Net and U-Net+ was performed. All the trainings were performed on two Nvidia GTX 1080 Ti GPUs, with Keras 2.2.4 (Tensorflow 1.12.0 backend) as framework.

### Predictions post-processing

During the inference phase, the image from test set was first pre-processed as “Images pre-processing” section shown and decomposed to multiple patches as “Training and validation set generation” section illustrated. As described above, when the height or width of image is indivisible by 256, overlapping patches will be generated at the region near the edge of image (see Fig. 4c). For the non-overlapping patch, the predicted value per pixel was determined to 1 (cell nuclei) or 0 (background) based on whether the output value of model at that pixel was larger than 0.5 or not. In contrast, to the overlapping part, the maximum value of all predictions from different patches at one pixel was first selected and then the label at that pixel was determined using the same rule as non-overlapping part (1 if the maximum value larger than 0.5, otherwise 0). Final masks were constructed after performing two simple morphological operations to remove the small holes and isolated objects with approximately area size of several hundred pixels.

### Metrics to evaluate the performance of model

As suggested in [21], selection of metrics plays pivotal role in evaluating the performance of deep learning models. In the experiments, average aggregated method was selected as the metric due to its strong statistically robustness [21]. We compared the intersection over union (IOU) (not the Dice coefficient in the training phase) cell by cell. Specifically, one ground truth and predicted image were first decomposed to multiple connected regions and each connected region represents one true cell or predicted cell respectively. For every cell in the ground truth set {*g*_*i*_,*i*=1,...,*N*_*g*_}, we iterated the predicted label set {*p*_*j*_,*j*=1,...,*N*_*p*_} and found one whose IOU value with *g*_*i*_ was greater than a preset threshold from {*T*_*k*_}. If such a predicted mask existed, it was considered as a true positive (TP) prediction (the IOU value was recorded), otherwise *g*_*i*_ was categorized as a false negative (FN) truth (the IOU value was set to 0.0 accordingly). In other words, each isolated cell in ground truth image will be assigned one value, which is either the IOU value with TP case or 0.0 with FN case. False positive (FP) value was counted when the IOU of one predicted cell with all *g*_*i*_ was 0.0. The average IOU could then be calculated for one image. Besides, we followed the precision definition in the contest, namely for one predicted image, the precision is defined as (similar as AP_50:95_ commonly employed in object detection task),

3 $$ \text{precision} = \frac{1}{|\{T_{k}\}|} \sum_{k} \frac{\text{TP}_{k}}{\text{TP}_{k} + \text{FN}_{k} + \text{FP}_{k}}  $$

In the equation, {*T*_*k*_} is the collection of values starting from 0.50 to 0.95 with step size 0.05. Eventually, the average IOU and precision for all the images in the test set were calculated and used as the metric values to evaluate the performance of algorithm (see “Results” section).

The average inference time was also evaluated using GPU (one Nvidia GTX 1080 Ti). In the experiments, the total inference time for all the images in test set (53 images, 174 patches) was counted and the above procedure was repeated for 5 times. Eventually the average inference time per patch (256×256) was estimated.

## Data Availability

The datasets generated and/or analyzed during the current study are available in 2018 Kaggle Data Science Bowl repository, https://www.kaggle.com/c/data-science-bowl-2018#evaluation.

## Abbreviations

CLAHE: Contrast limited adaptive histogram equalization
CNN: Convolutional neural network
DL: Deep learning
FN: False negative
FP: False positive
FPN: Feature pyramid network
IOU: Intersection over union
MRI: Magnetic resonance imaging
RBC: Red blood cell
ReLU: Rectified linear unit
TP: True positive
WBC: White blood cell

## Footnotes

1 code from https://github.com/zhixuhao/unet

2 code from https://github.com/MrGiovanni/UNetPlusPlus
